# Supplementary material for: Integrated systems approach defines the antiviral pathways conferring protection by the RV144 HIV vaccine
Source: Nat Commun. 2019 Feb 20;10:863. doi: 10.1038/s41467-019-08854-2 (PMC6382801; doi:10.1038/s41467-019-08854-2)
Supplement: Supplementary file 1 — Supplementary Information [file 41467_2019_8854_MOESM1_ESM.pdf]

Supplementary Information

Integrated systems approach defines the antiviral pathways conferring protection by the RV144 HIV vaccine

Fourati et al.

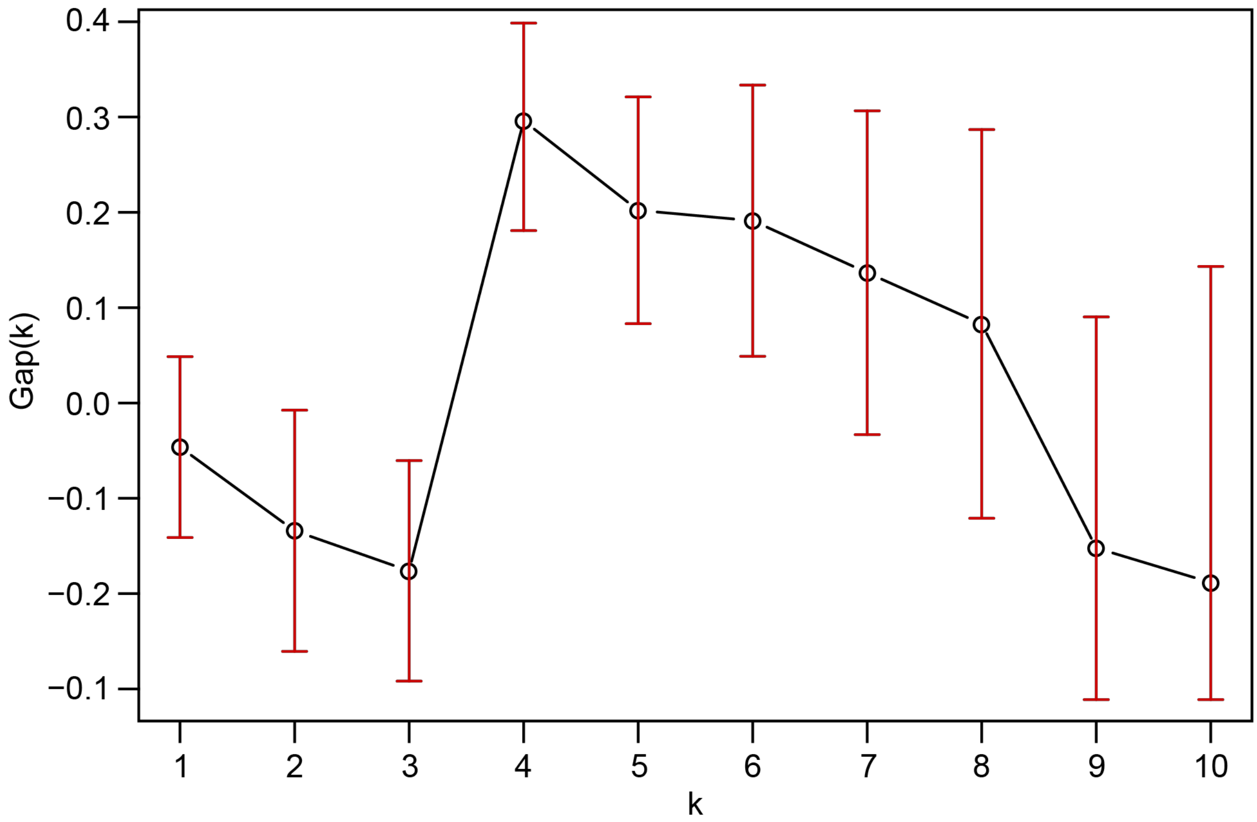

Supplementary Fig. 1. Gap statistic revealed four clusters of correlated genesets. Scatter plot showing the gap statistic estimation of the optimal number of cluster of genes among the 11 pathways induced by the RV144 vaccine in the transcriptomic pilot cohort. The x--axis corresponds to the different number of clusters tested and the y--axis corresponds to the gap statistic (Gap) and its 95% confidence interval calculated over 100 bootstrap iterations. The gap statistic corresponds to the between- clusters variance divided by the intra-cluster variance (the greater the gap statistic, the better the fit). The optimal number of clusters was obtained following the rule described in Tibshirani *et al.*[1] defined as the smallest k such that $Gap(k) \geq Gap(k+1) - sd(k+1)$. Consequently, four clusters were identified as the optimal number of clusters of pathways.

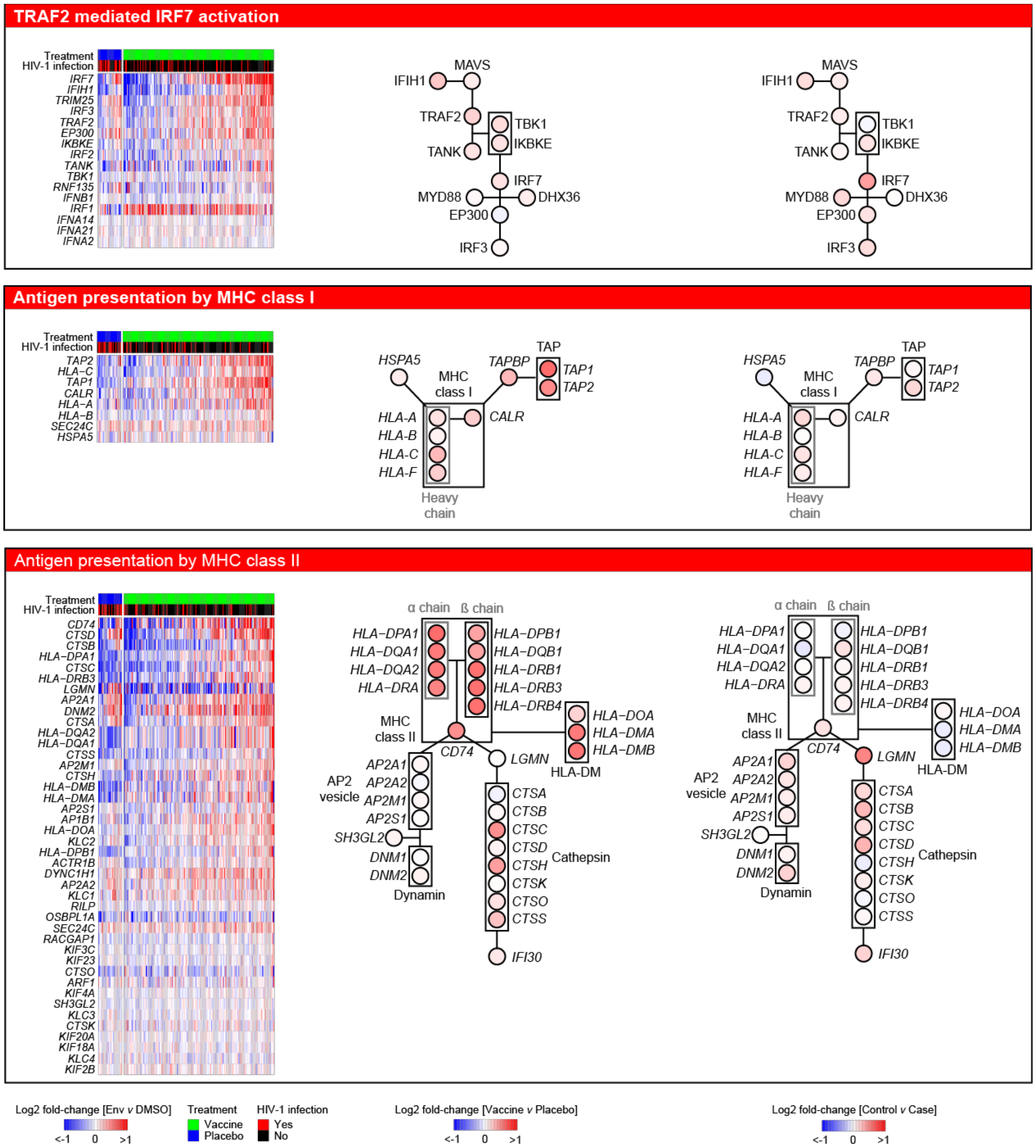

Supplementary Fig. 2. IRF7 activation, MHC class I/II are induced by the vaccine and associated with low risk of HIV-1 acquisition. GSEA was used to identify canonical pathways (MSigDB v.5.1, class C2P) enriched in genes induced by the RV144 vaccine and up-regulated in participants that remained HIV-1 negative at last follow-up (Control) compared to participants that acquired HIV-1 despite being vaccinated (Case). Three canonical pathways, TRAF2 activation of IRF7 (upper panel), Antigen presentation by MHC class I (middle panel) and Antigen presentation by MHC class II (lower panel) are presented. For each panel, a heatmap showing the expression of the leading edges genes induced by the vaccine or induced in controls versus cases is presented on the left. A network based on interactions (edges) described in the literature (Reactome, Literome) with each node (genes) colored by the fold-change between vaccine and placebo (middle of the panel) and by the fold-change between controls versus cases (right side) is given for each canonical pathway.

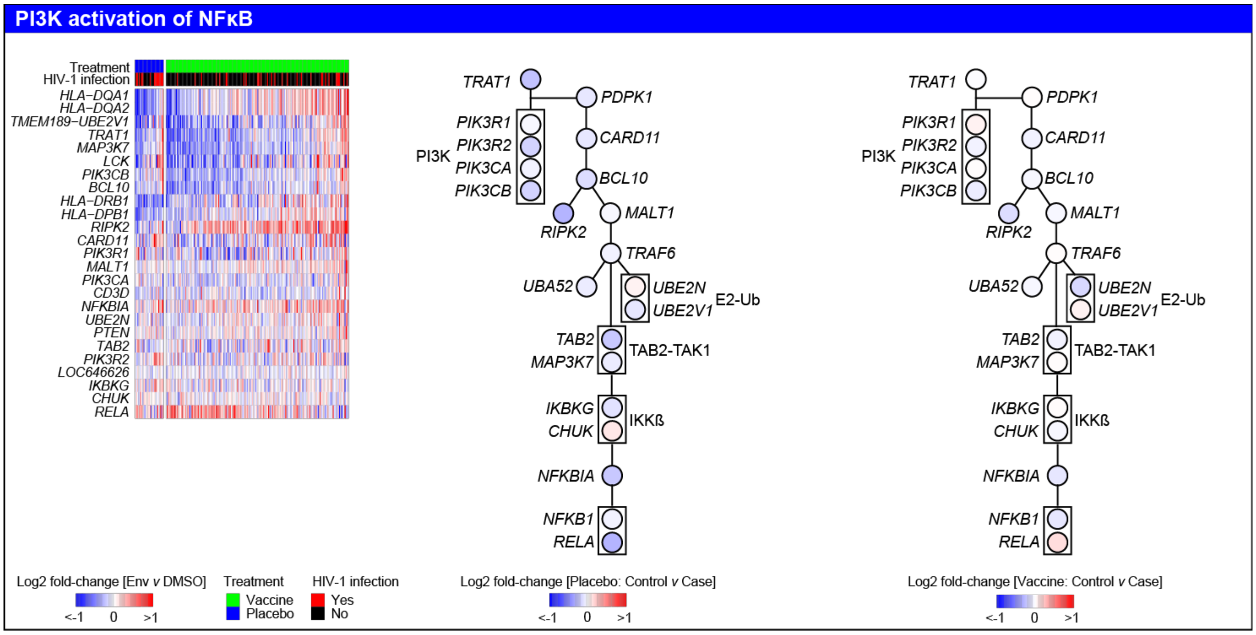

Supplementary Fig. 3. NF-κB activation is associated with HIV-1 acquisition in placebo and vaccinees. GSEA was used to identify canonical pathways (MSigDB v.5.1, class C2P) enriched in genes down-regulated in participants that remained HIV-1 negative at last follow-up (Control) compared to participants that acquired HIV-1 (Case), for both placebo recipient and RV144 vaccinees. One canonical pathway, the NF-κB activation is shown in the figure. A heatmap presenting the expression of the leading edges genes induced in cases versus controls is displayed on the left side. A network based on interactions (edges) described in the literature (Reactome, Literome) with each node (genes) of the network colored by the fold-change between controls versus cases in placebo recipient (middle of the panel) and vaccinees (right side) is given for the canonical pathway.

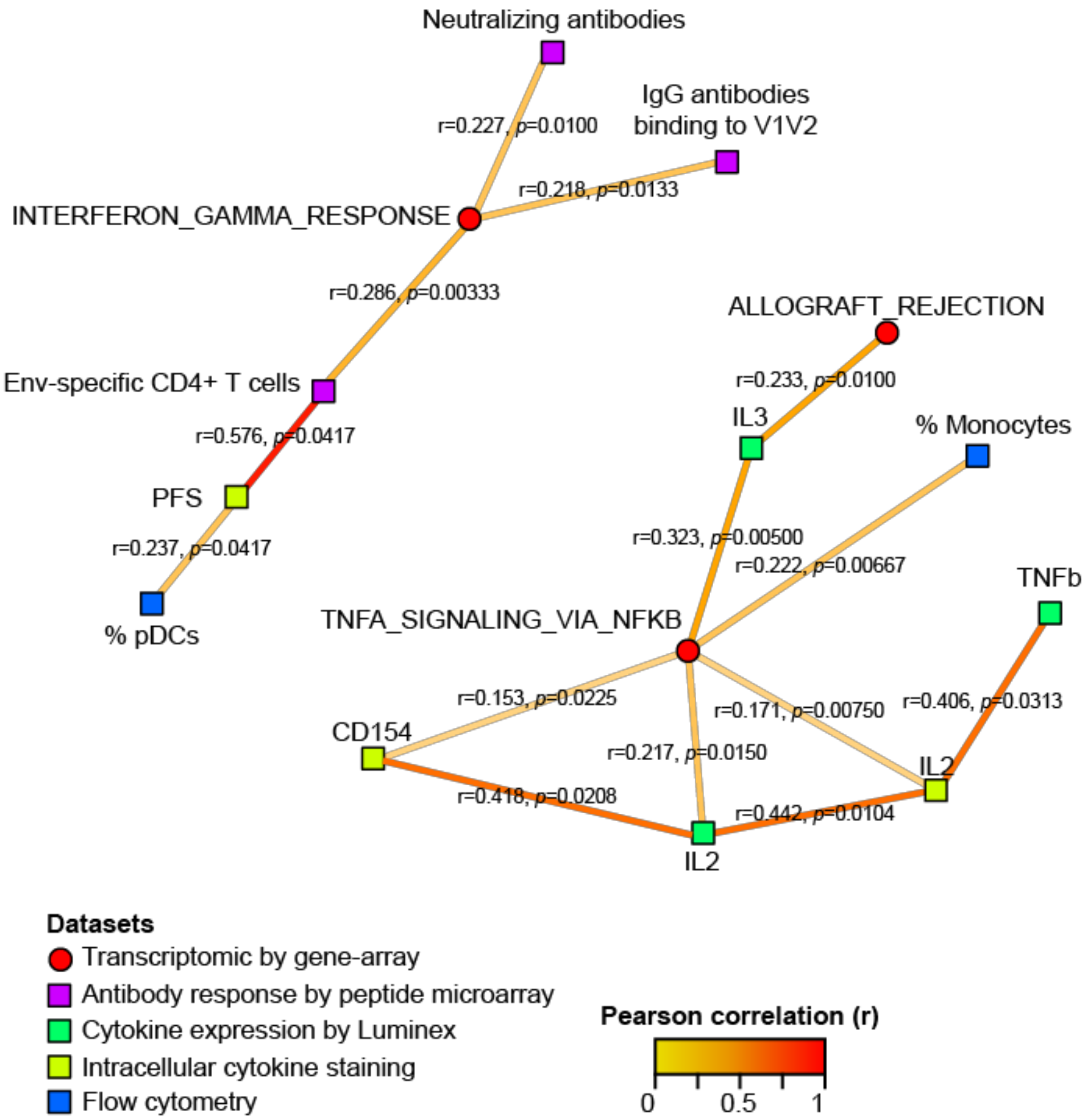

Supplementary Fig. 4. Integrative analysis of the transcriptomic, antibody response, and cytokine expression. The RV144 vaccine modulated four pathways (Hallmark pathways identified using the Gene Set Enrichment Analysis[2]). A projection-based integrative analysis[3] was performed to determine associations between those four pathways and previously investigated readouts. Each dot corresponds to a readout; edges connect two readouts significantly positively correlated (Pearson t-test: $p \leq 0.05$). Each edge is labeled with the Pearson correlation and its corresponding p-value. Among the four pathways modulated by the RV144 vaccine, three pathways (red circle) were significantly correlated to immune subset frequencies, antibody or cytokine expression (squares). IFNγ signaling pathway associated with a low risk of HIV-1 acquisition of RV144 vaccinees (Interferons pathways) were significantly correlated to levels of IgG against V1/V2, a previously identified marker of good response to the RV144 vaccine. TNF_{α} signaling pathway associated with a higher risk of HIV-1 acquisition was significantly positively correlated with inflammatory cytokines IL2+IL3 and the frequency of monocytes (inflammatory effector cells).

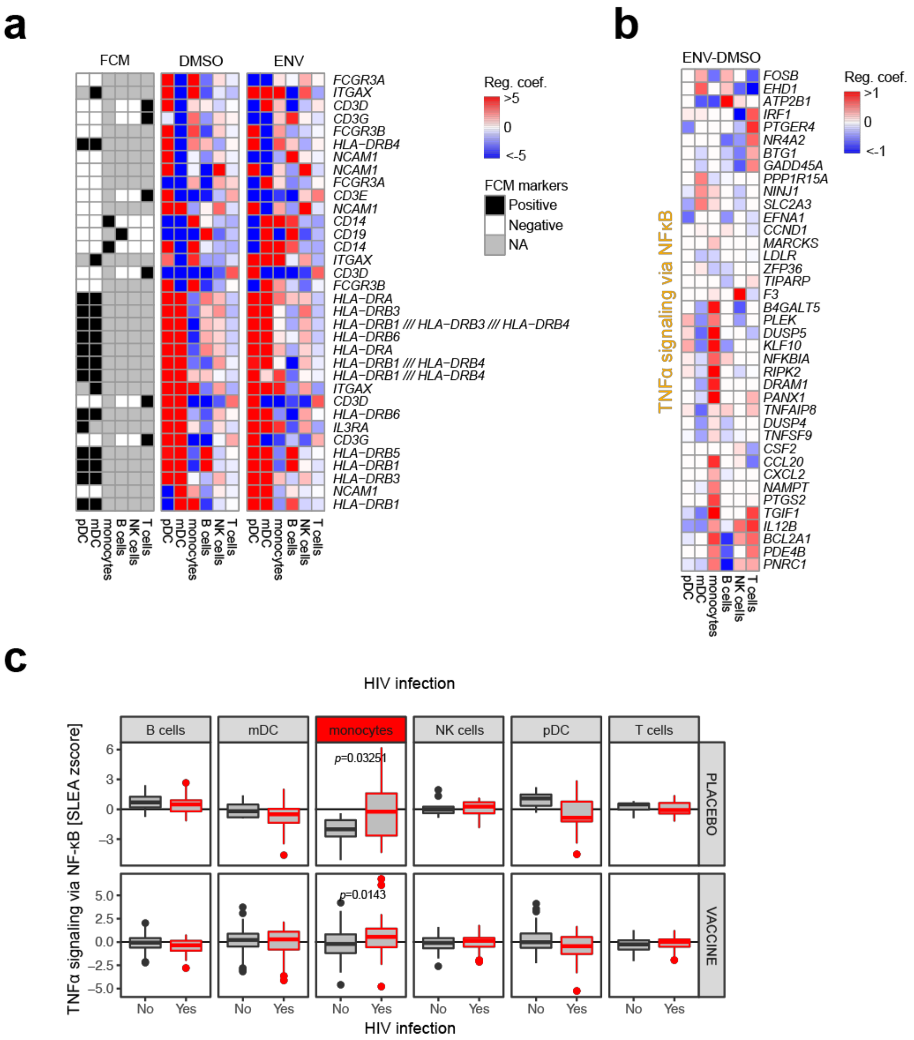

Supplementary Fig. 5. Deconvolution of PBMC expression. **a** Heatmap presenting the expression of genes coding for cells surface markers in the deconvoluted matrix. **b** Heatmap showing the expression of the 40 NF-κB target genes in the six deconvoluted subsets. For each gene a linear model was fit and deconvolution was applied. The regression coefficient (*i.e.* the cell-specific expression for each gene) is plotted using a blue-white-red color gradient. The monocytes subsets expressed the highest levels of the NF-κB target genes compared to the other subsets (Wilcoxon rank-sum test: p=1.64e-06). **c** Boxplots of NF-κB target genes in the six subsets stratified by HIV-1 infection. A Wilcoxon rank-sum test was performed to assess the difference between participants that acquire HIV-1 and those that remained HIV-1 negative. On the boxplot, the lower whisker, the lower hinge, the middle hinge, the upper hinge and the upper whisker correspond to the interquartile (IQR) from the 1st quartile, the 1st quartile, the median, the 3rd quartile and the IQR from the 3rd quartile, respectively.

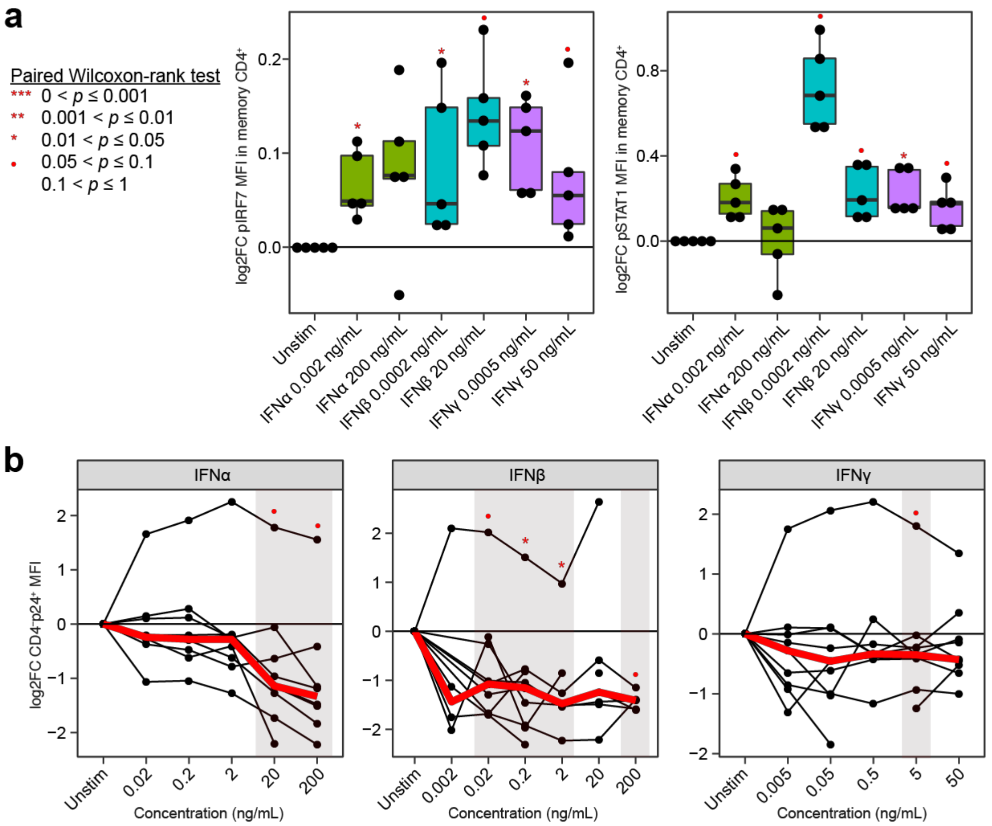

Supplementary Fig. 6. Treatment of lymphocytes with interferons result in the activation of IRF7 and render them refractory to infection. **a** Boxplot of the ratio of the phosphorylated IRF7 and phosphorylated STAT1 after interferon stimulation of memory CD4+ compared to unstimulated memory CD4+. The *ex vivo* experiments were performed on cells from five healthy donors. The fold-change in median of fluorescence intensity (MFI) between interferon stimulated samples and the unstimulated condition is presented as a function of the concentration of interferon used. On the boxplot, the lower whisker, the lower hinge, the middle hinge, the upper hinge and the upper whisker correspond to the interquartile (IQR) from the 1st quartile, the 1st quartile, the median, the 3rd quartile and the IQR from the 3rd quartile, respectively. **b** Lines plot showing the ratio of $CD4^{-}p24^{+}$ MFI after interferon stimulation over the unstimulated levels as a function of interferon concentration. The red line indicates the median levels of $CD4^{-}p24^{+}$ MFI across 10 healthy donors. **a-b** A paired Wilcoxon rank-sum test was used to assess the statistical significance of the fold-change (***: $p \leq 0.001$, **: $0.001 < p \leq 0.01$, *: $0.01 < p \leq 0.05$, •: $0.05 < p \leq 0.1$).

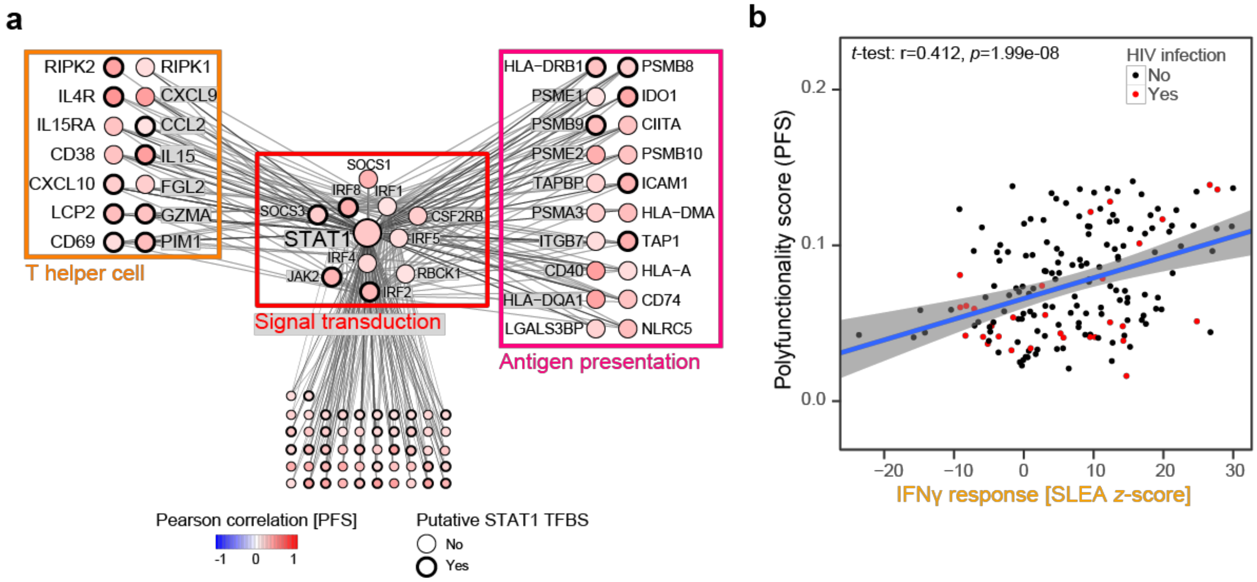

Supplementary Fig. 7. Mechanisms associated with an increased CD4+ T cell response. **a** Network showing the genes implicated in IFNγ signaling. Nodes correspond to genes; the color of a node is proportional to the Pearson correlation with Polyfunctionality score (PFS). Edges are inferred by GeneMANIA and correspond to physical interactions, colocalization or co-expression. **b** Scatter plot presenting the association of IFNγ responsive genes as a function of the levels of PFS. The average expression of the list of genes calculated using the SLEA z-score method is presented on the x-axis. A linear regression model (blue line), and its 95% confidence interval (grey zone), was fit between SLEA z-score and PFS. A Pearson correlation and a t-test were performed to assess the significance of the correlation between the transcriptomic data and CD4+ T cell response.

Supplementary Table 1. Clinical characteristics of the RV144 cohort and transcriptomic pilot study

	Full cohort	Transcriptomic pilot study	Fisher exact test: *p*
n	16,402	50	
Treatment			2.33e-05
vaccine	8,202 (50.0%)	40 (80%)	
placebo	8,200 (50.0%)	10 (20%)	
HIV infection			0.765
acquired	132 (0.805%)	0 (0%)	
negative	15,823 (96.5%)	50 (100%)	
N/A	447 (2.73%)	0 (0%)	
Gender			0.110
male	10,068 (61.4%)	25 (50%)	
female	6,334 (38.6%)	25 (50%)	
Age group			0.166
≤ 20 yr	4,546 (27.7%)	16 (32%)	
21-25 yr	7,344 (44.8%)	16 (32%)	
≥ 26 yr	4,512 (27.5%)	18 (36%)	
Province			0.575

Chon Buri	8,219 (50.1%)	23 (46%)	
Rayong	8,183 (49.9%)	27 (54%)	
Risk group			0.884
low	7,789 (47.5%)	22 (44%)	
medium	4,664 (28.4%)	15 (30%)	
high	3,949 (24.1%)	13 (26%)	
Per-protocol			2.25e-06
yes	12,542 (76.5%)	50 (100%)	
no	3,860 (23.5%)	0 (0%)	

Risk group: Risk of HIV-1 infection based on participants behavior; Per protocol: completed the full immunization course (4/4 immunizations).

Supplementary Table 2. Clinical characteristics of the placebo and vaccinees of the transcriptomic pilot study

	Vaccine	Placebo	Fisher exact test: *p*
n	40	10	
HIV infection			
controls	40 (100%)	10 (100%)	
Gender			1
male	20 (50%)	5 (50%)	
female	20 (50%)	5 (50%)	
Age group			0.907
≤ 20 yr	12 (30.0%)	4 (40%)	
21-25 yr	13 (32.5%)	3 (30%)	
≥ 26 yr	15 (37.5%)	3 (30%)	
Province			0.0850
Chon Buri	21 (52.5%)	2 (20%)	
Rayong	19 (47.5%)	8 (80%)	
Risk group			0.189
low	19 (47.5%)	3 (30%)	
medium	13 (32.5%)	2 (20%)	
high	8 (20.0%)	5 (50%)	
Per-protocol			
yes	40 (100%)	10 (100%)	

Risk group: Risk of HIV-1 infection based on participants behavior; Per protocol: completed the full immunization course (4/4 immunizations).

Supplementary Table 3. Clinical characteristics of the RV144 cohort and transcriptomic case/control study

	Full cohort	Transcriptomic case/control study	Fisher exact test: *p*
n	16,402	213	
Treatment			< 2.2e-16
vaccine	8,202 (50.0%)	183 (85.9%)	
placebo	8,200 (50.0%)	30 (14.1%)	

HIV infection			< 2.2e-16
acquired	132 (0.805%)	48 (22.5%)	
negative	15,823 (96.5%)	165 (77.5%)	
N/A	447 (2.73%)		
Gender			0.671
male	10,068 (61.4%)	134 (62.9%)	
female	6,334 (38.6%)	79 (37.1%)	
Age group			0.385
≤ 20 yr	4,546 (27.7%)	52 (24.4%)	
21-25 yr	7,344 (44.8%)	105 (49.3%)	
≥ 26 yr	4,512 (27.5%)	56 (26.3%)	
Province			0.581
Chon Buri	8,219 (50.1%)	111 (52.1%)	
Rayong	8,183 (49.9%)	102 (47.9%)	
Risk group			0.746
low	7,789 (47.5%)	98 (46.0%)	
medium	4,664 (28.4%)	59 (27.7%)	
high	3,949 (24.1%)	56 (26.3%)	
Per-protocol			2.83E-06
yes	12,542 (76.5%)	190 (89.2%)	
no	3,860 (23.5%)	23 (10.8%)	

Risk group: Risk of HIV-1 infection based on participants behavior; Per protocol: completed the full immunization course (4/4 immunizations).

Supplementary Table 4. Clinical characteristics of the placebo and vaccines of the transcriptomic case/control study

	Vaccine	Placebo	Fisher's exact test: *p*
n	183	30	
HIV infection			
acquired	31 (16.9%)	17 (56.7%)	1.05e-05
negative	152 (83.1%)	13 (43.4%)	
Gender			
male	117 (63.9%)	17 (56.7%)	
female	66 (36.1%)	13 (43.3%)	
Age group			
≤ 20 yr	44 (24.1%)	8 (26.7%)	0.966
21-25 yr	91 (49.7%)	14 (46.6%)	
≥ 26 yr	48 (26.2%)	8 (26.7%)	
Province			0.114
Chon Buri	91 (49.7%)	20 (66.7%)	
Rayong	92 (50.3%)	10 (33.3%)	
Risk group			
low	84 (45.9%)	14 (46.7%)	0.651
medium	49 (26.8%)	10 (33.3%)	
high	50 (27.3%)	6 (20.0%)	

Per-protocol			0.0508
yes	160 (87.4%)	30 (100%)	
no	23 (12.6%)	0 (0%)	

Risk group: Risk of HIV-1 infection based on participants behavior; Per protocol: completed the full immunization course (4/4 immunizations).

Supplementary Table 5. Associated of the four representative pathways with HIV acquisition

Geneset	Placebo NES	Placebo q-value	Vaccine NES	Vaccine q-value
HALLMARK_TNFA_SIGNALING_VIA_NFKB	-2.17	**0**	-2.21	**0**
HALLMARK_MTORC1_SIGNALING	-1.85	**0.000860**	-1.70	**0.00893**
HALLMARK_INTERFERON_GAMMA_RESPONSE	-1.42	0.0645	1.61	**0.0281**
HALLMARK_ALLOGRAFT_REJECTION	-0.893	0.785	-1.14	0.320

Two pathways are significantly associated with HIV acquisition (GSEA: FDR ≤ 5%) while one pathway is significantly associated with protection from HIV acquisition.

Supplementary Table 6. Univariate and multivariate analysis of IgG response and IFN signaling among vaccinees

	Univ.OR (95% CI)	Univ. *p*	Multiv. OR (95% CI)	Multiv. *p*
IgG antibodies binding to V1/V2	0.703 [0.446, 1.07]	0.112	1.22 [0.728, 2.03]	0.443
DPB1*13	0.696 [0.270, 1.64]	0.426	0.406 [0.0890, 1.25]	0.165
Interaction IgG:DPB1*13	---	---	0.111 [0.0187, 0.427]	**0.00472**
INTERFERON_GAMMA_RESPONSE	0.883 [0.803, 0.967]	**0.00837**	0.898 [0.811, 1.01]	0.0542

For each variable, the odds ratio (OR) of HIV-1 acquisition and its 95% confidence interval (CI) is reported per one standard deviation increase. The p-value of a z-test, testing that the OR is different from 1 is reported in the table. P-values inferior or equal 0.05 are indicated in bold. All univariate (univ.) and multivariate (multiv.) logistic regression models were adjusted for gender and behavior risk of the participants.

Supplementary References

1. Tibshirani, R., Walther, G. & Hastie, T. Estimating the number of clusters in a data set via the gap statistic. *J. R. Stat. Soc. Ser. B Stat. Methodol.* (2001).
2. Subramanian, A. *et al.* Gene set enrichment analysis: a knowledge-based approach for interpreting genome-wide expression profiles. *Proc Natl Acad Sci U S A* **102**, 15545–15550 (2005).
3. Rohart, F., Gautier, B., Singh, A. & Lê Cao, K.-A. mixOmics: An R package for ‘omics feature selection and multiple data integration. *PLOS Comput. Biol.* **13**, e1005752 (2017).
