# Supplementary material for: Integrated systems approach defines the antiviral pathways conferring protection by the RV144 HIV vaccine
Source: Nat Commun. 2019 Feb 20;10:863. doi: 10.1038/s41467-019-08854-2 (PMC6382801; doi:10.1038/s41467-019-08854-2)
Supplement: Supplementary file 3 — Description of Additional Supplementary Files [file 41467_2019_8854_MOESM3_ESM.pdf]

Description of Additional Supplementary Files

File Name: Supplementary Data 1
Description: Pathways modulated by Env stimulation and different between RV144 vaccinees and placebo recipients Before modeling the gene-expression using LIMMA and identifying pathways modulated by the RV144 vaccine, the DMSO condition was subtracted from the Env stimulated gene-expression, followed by the subtraction of the pre-vaccination condition from the week 26 expression. The resulting expression matrix was then used to identify genesets significantly associated with the difference between RV144 vaccinees and placebo recipients (MSigDB: Hallmark; GSEA: FDR ≤ 5%). The leading edges genes of each geneset significantly associated with response to the RV144 vaccine are provided in the table. The four representative pathways associated with the response to the RV144 vaccine are highlighted in red.

File Name: Supplementary Data 2
Description: Differential expression of leading edge genes of the four representative pathways associated with the response to the RV144 vaccine For each gene, its corresponding probe on the beadarray (lmnID), the log2 fold-change (log2FC) between vaccinees and placebo recipients corrected for pre-vaccination expression, moderated ttest p-value (P.Value) and the Benjamini-Hochberg corrected p-value (adj.P.Val) are given. Genes highlighted in black are also associated with HIV-1 acquisition in the RV144 case/control cohort (Supplementary Data 5).

File Name: Supplementary Data 3
Description: Canonical pathways, putative transcription factor targets and immune cell markers overlapping the four representative pathways associated with the response to the RV144 vaccine List of canonical pathways (MSigDB: C2P), putative transcription factor targets (MSigDB: C3TF) and immune cell markers48 overlapping the four representative pathways associated with the response to the RV144 vaccine (Jaccard Index ≥ 0.25 or Fisher exact test: p ≤ 0.1). For each overlapping pathway, its enrichment among genes induced by the RV144 vaccine (GSEA: NES) and the corresponding false discovery rate (GSEA: FDR q-val), as well as their leading edges genes (LEADING_EDGE) and the leading edges genes overlapping the four representative genesets are provided in the table.

File Name: Supplementary Data 4
Description: Functional annotation of the leading edges genes using REACTOME and GeneRIF

File Name: Supplementary Data 5
Description: . List of leading edges of the 3 genesets significantly associated with HIV acquisition in vaccinees For each geneset, the list of leading edge genes and the functional description of those genes is provided.

File Name: Supplementary Data 6
Description: Canonical pathways, putative transcription factor targets and immune cell markers overlapping significantly the 3 genesets significantly associated with HIV acquisition in vaccinees List of canonical pathways (MSigDB: C2P), putative transcription factor targets (MSigDB: C3TF) and immune cell markers48 overlapping significantly (Jaccard Index ≥ 0.25 or Fisher exact test: p ≤ 0.1) the 3 genesets significantly associated with HIV acquisition in vaccinees. For each overlapping pathway, its enrichment among genes induced by the RV144 vaccine (GSEA: NES) and the corresponding false discovery rate (GSEA: FDR q-val), as well as their leading edge genes (LEADING_EDGE) and the leading edges genes overlapping the 3 genesets significantly associated with HIV acquisition in vaccines.

File Name: Supplementary Data 7
Description: Functional annotation of the leading edges genes using REACTOME and GeneRIF An asterisk (*) indicates genes part of the REACTOME_IMMUNE_SYSTEM geneset significantly overlapping the HALLMARK_INTERFERON_GAMMA_RESPONSE geneset (Supplementary Data 6)

File Name: Supplementary Data 8
Description: List of markers included in the integrative analysis

File Name: Supplementary Data 9
Description: Two of the four representative pathways induced by the RV144 vaccine were associated with antibody response among RV144 vaccinees Pathways significantly associated with antibody response (GSEA: FDR ≤ 5%) are provided in the table.

File Name: Supplementary Data 10
Description: List of the leading edges of the two genesets significantly associated with antibody response in vaccinees For each geneset, the list of leading edge genes and the functional description of those genes is provided.

File Name: Supplementary Data 11
Description: Canonical pathways, putative transcription factor targets and immune cell markers overlapping significantly the two representative pathways associated with antibody response in RV144 vaccinees List of canonical pathways (MSigDB: C2P), putative transcription factor targets (MSigDB: C3TF) and immune cell markers48 overlapping significantly (Jaccard Index ≥ 0.25 or Fisher exact test: p ≤ 0.1) the two representative pathways associated with antibody response in RV144 vaccinees. For each overlapping pathway, its enrichment among genes induced by the RV144 vaccine (GSEA: NES) and the corresponding false discovery rate (GSEA: FDR q-val), as well as their leading edges genes (LEADING_EDGE) and the leading edges genes overlapping the two representative pathways associated with antibody response in RV144 vaccinees.

File Name: Supplementary Data 12
Description: Functional annotation of the leading edges genes using REACTOME and GeneRIF

File Name: Supplementary Data 13
Description: IFNγ response induced by the RV144 vaccine was associated with cellular response among RV144 vaccinees IFNγ response induced by the RV144 vaccine was associated with cellular response (PFS) among RV144 vaccinees (GSEA: FDR ≤ 5%)

File Name: Supplementary Data 14
Description: List of the leading edges of the IFNγ response associated with cellular response in vaccinees The list of the leading edge genes and the functional description of those genes is provided.

File Name: Supplementary Data 15
Description: Functional annotation of the IFNγ response leading edges genes using REACTOME and GeneRIF
