# Supplementary material for: Integrated systems approach defines the antiviral pathways conferring protection by the RV144 HIV vaccine
Source: Nat Commun. 2019 Feb 20;10:863. doi: 10.1038/s41467-019-08854-2 (PMC6382801; doi:10.1038/s41467-019-08854-2)
Supplement: Supplementary file 19 — Reporting Summary [file 41467_2019_8854_MOESM19_ESM.pdf]

nature research

Corresponding author(s): Rafick-Pierre Sekaly

Last updated by author(s): Dec 21, 2018

Reporting Summary

Nature Research wishes to improve the reproducibility of the work that we publish. This form provides structure for consistency and transparency in reporting. For further information on Nature Research policies, see Authors & Referees and the Editorial Policy Checklist.

Statistics

For all statistical analyses, confirm that the following items are present in the figure legend, table legend, main text, or Methods section.

n/a	Confirmed	
☐	☒	The exact sample size (n) for each experimental group/condition, given as a discrete number and unit of measurement
☐	☒	A statement on whether measurements were taken from distinct samples or whether the same sample was measured repeatedly
☐	☒	The statistical test(s) used AND whether they are one- or two-sided<br>*Only common tests should be described solely by name; describe more complex techniques in the Methods section.*
☐	☒	A description of all covariates tested
☐	☒	A description of any assumptions or corrections, such as tests of normality and adjustment for multiple comparisons
☐	☒	A full description of the statistical parameters including central tendency (e.g. means) or other basic estimates (e.g. regression coefficient) AND variation (e.g. standard deviation) or associated estimates of uncertainty (e.g. confidence intervals)
☐	☒	For null hypothesis testing, the test statistic (e.g. F, t, r) with confidence intervals, effect sizes, degrees of freedom and P value noted<br>*Give P values as exact values whenever suitable.*
☒	☐	For Bayesian analysis, information on the choice of priors and Markov chain Monte Carlo settings
☐	☒	For hierarchical and complex designs, identification of the appropriate level for tests and full reporting of outcomes
☐	☒	Estimates of effect sizes (e.g. Cohen's d, Pearson's r), indicating how they were calculated

Our web collection on statistics for biologists contains articles on many of the points above.

Software and code

Policy information about availability of computer code

Data collection	*Provide a description of all commercial, open source and custom code used to collect the data in this study, specifying the version used OR state that no software was used.*
Data analysis	All data analysis was performed using R language and source code to generate the figures of the article is provided on sekalylab github page (https://github.com/sekalylab/rv144)

For manuscripts utilizing custom algorithms or software that are central to the research but not yet described in published literature, software must be made available to editors/reviewers. We strongly encourage code deposition in a community repository (e.g. GitHub). See the Nature Research guidelines for submitting code & software for further information.

Data

Policy information about availability of data

All manuscripts must include a data availability statement. This statement should provide the following information, where applicable:
- Accession codes, unique identifiers, or web links for publicly available datasets
- A list of figures that have associated raw data
- A description of any restrictions on data availability

The microarray data have been submitted to GEO: GSE103740. All other data supporting the findings of this study are available from the authors upon request.

Field-specific reporting

Please select the one below that is the best fit for your research. If you are not sure, read the appropriate sections before making your selection.

☒ Life sciences ☐ Behavioural & social sciences ☐ Ecological, evolutionary & environmental sciences

Life sciences study design

All studies must disclose on these points even when the disclosure is negative.

Sample size	A power analysis was performed to assess the association of a candidate marker and risk of HIV-1 acquisition the RV144 vaccine [Haynes BF et al., 2012 N Engl J Med]. The same cohort (i.e. same sample size) was used our article.
Data exclusions	RV144 participants that didn't complete the full vaccination regimen or that acquired HIV before the completion of the vaccination were excluded. Patients with missing plasma and PBMC samples were excluded. Full exclusion criteria are in Haynes BF et al., 2012 N Engl J Med.
Replication	No technical replicates. Transcriptomic datasets included >30 biological replicates (independent participants). HIV-1 infectability assays were performed using PBMC from 10 healthy donors (biological replicates).
Randomization	The initial RV144 clinical trial was a double-blind placebo-controled randomize clinical trial. Randomization is not applicable for the transcriptomic analysis.
Blinding	During the generation of the transcriptomic datasets, we were blinded of the treatment groups (vaccine or placebo) and outcome (acquisition of HIV). The bioinformatic analysis was first performed blinded, then only with the treatment group information (2nd round of analysis) and finally with all clinical information (3rd round of analysis).

Reporting for specific materials, systems and methods

We require information from authors about some types of materials, experimental systems and methods used in many studies. Here, indicate whether each material, system or method listed is relevant to your study. If you are not sure if a list item applies to your research, read the appropriate section before selecting a response.

Materials & experimental systems

n/a	Involved in the study	
☐	☒	Antibodies
☒	☐	Eukaryotic cell lines
☒	☐	Palaeontology
☒	☐	Animals and other organisms
☐	☒	Human research participants
☐	☒	Clinical data

Methods

n/a	Involved in the study	
☒	☐	ChIP-seq
☒	☐	Flow cytometry
☒	☐	MRI-based neuroimaging

Antibodies

Antibodies used	All antibodies used for intracellular cytokine staining and Flow cytometry were puchased from BD. This includes CD4–fluorescein isothiocyanate (FITC), CD3-allophycocyanin (APC), IFNγ-phycoerythrin (PE), Il-2-phycoerythrin (PE) and CD8-PerCP-Cy5.5.
Validation	*Describe the validation of each primary antibody for the species and application, noting any validation statements on the manufacturer's website, relevant citations, antibody profiles in online databases, or data provided in the manuscript.*

Human research participants

Policy information about studies involving human research participants

Population characteristics	RV144 trial is community-based clinical trial done in Thailand. Thai men and women who were between the ages of 18 and 30 years and who were not in- fected with HIV were recruited from the com- munity without regard to HIV risk (i.e., commu- nity risk).
Recruitment	The trial was conducted through facilities of the Thai Ministry of Public Health in Rayong and Chon Buri provinces. From September 2003 through December 2005 were volonteers were asked to fill a recruitment form (c.f. Rerks-Ngarm S et al. (2009) N Engl J Med). All participants of the transcriptomic study were part of the initial RV144 clinical trial.
Ethics oversight	The protocol was reviewed by the ethics committees of the Ministry of Public Health, the Royal Thai Army, Mahidol University, and the Human Subjects Research Review Board of the U.S. Army Medical Research and Materiel Command. It was also independently reviewed and endorsed by the World Health Organization and the Joint United Nations Program on HIV/AIDS and by the AIDS Vaccine Research Working Group of the National Institute of Allergy and Infectious Diseases at the National Institutes of Health.

Note that full information on the approval of the study protocol must also be provided in the manuscript.

Clinical data

Policy information about clinical studies

All manuscripts should comply with the ICMJE guidelines for publication of clinical research and a completed CONSORT checklist must be included with all submissions.

Clinical trial registration	ClinicalTrials.gov number: NCT00223080
Study protocol	Full protocol is described in Rerks-Ngarm S et al. (2009) N Engl J Med
Data collection	Data collection list and schedule is described in Rerks-Ngarm S et al. (2009) N Engl J Med
Outcomes	HIV-1 acquisition was the primary outcome of the RV144 clinical trial (and transcriptomic study).
